# Echocardiographic features of a giant aneurysm of membranous ventricular septum

**DOI:** 10.1186/s13019-024-02882-w

**Published:** 2024-06-21

**Authors:** Guobing Hu, Zhen Lv, Jie Ding, Ishaq Muhammad, Xiangming Zhu

**Affiliations:** Department of Ultrasound, the First Affiliated Hospital of Wannan Medical College, Wuhu, Anhui 241001 China

**Keywords:** Echocardiography, Aneurysm, Membranous ventricular septum

## Abstract

We describe the echocardiographic features of a 22-year-old female with a giant aneurysm of membranous ventricular septum (AMVS). Both transthoracic echocardiography (TTE) and transesophageal echocardiography (TEE) demonstrated significant dilatation of the aortic annulus and severe aortic regurgitation. A giant aneurysm was detected extending from a large membranous ventricular septal defect (MVSD) to the anterior surface of the aortic root. Contrast-enhanced CT and three-dimensional CT revealed a giant aneurysm located below the aortic root and connected to the left ventricular outflow tract (LVOT). The diagnosis was confirmed by surgery and postoperative pathological examination.

**Supplementary Information:**

The online version contains supplementary material available at 10.1186/s13019-024-02882-w.

## Case presentation

**Fig. 1:**
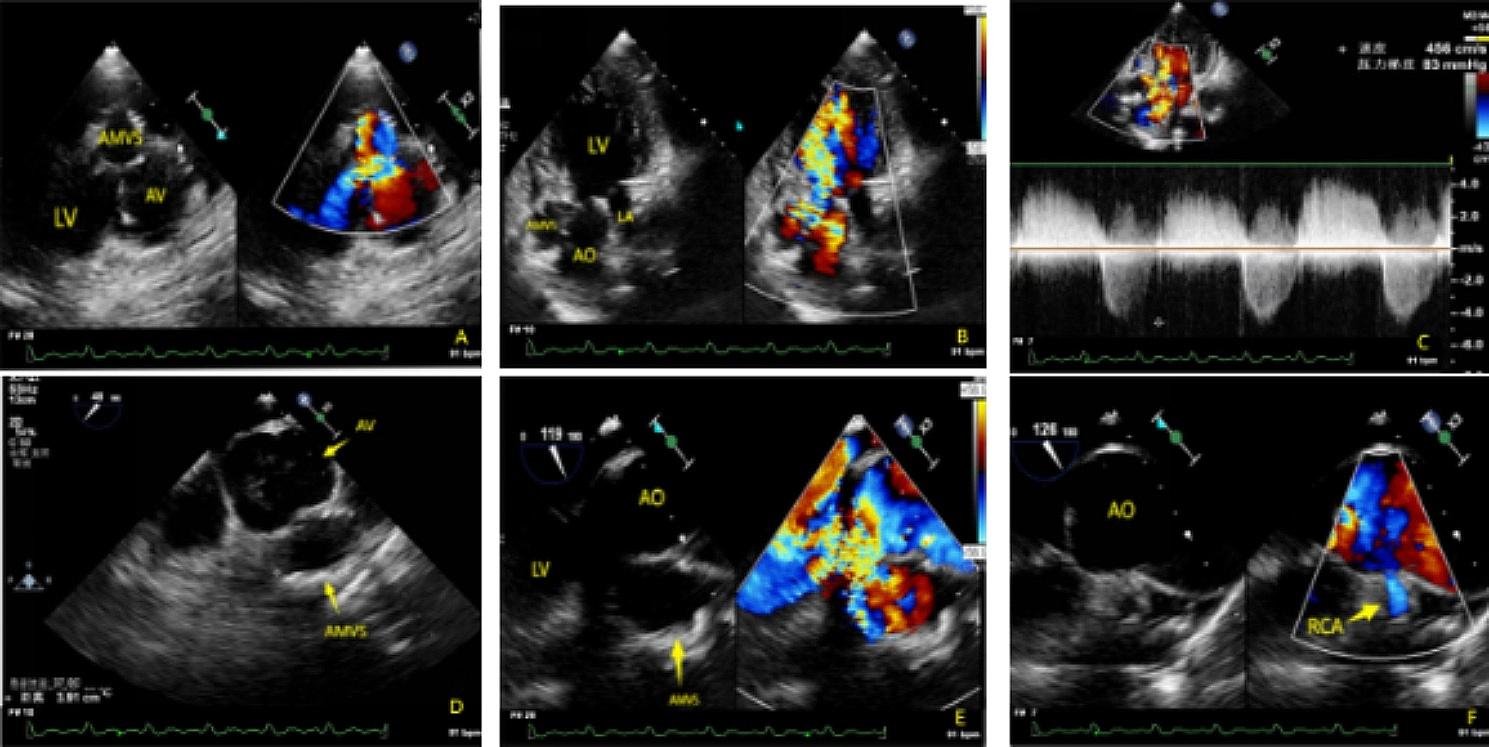
**(A)** TTE showed membranous ventricular septal defect (MVSD) with membranous ventricular septal aneurysm formed, and vortex blood flow signals were detected in the aneurysm during diastole. **(B)** TTE showed severe aortic valve regurgitation. **(C)** The systolic blood flow of the aortic valve was significantly increased, with a peak flow rate of about 4.56 m/s and a peak pressure gradience of about 83mmHg. **(D)** The aneurysm was located below the aortic root. **(E)** CDFI showed no significant blood flow signal into the MVSD during systolic, but a bright severe aortic regurgitation signal entering the aneurysm during diastole. **(F)** TEE showed that the aneurysm was close to the right coronary artery

**Fig. 2:**
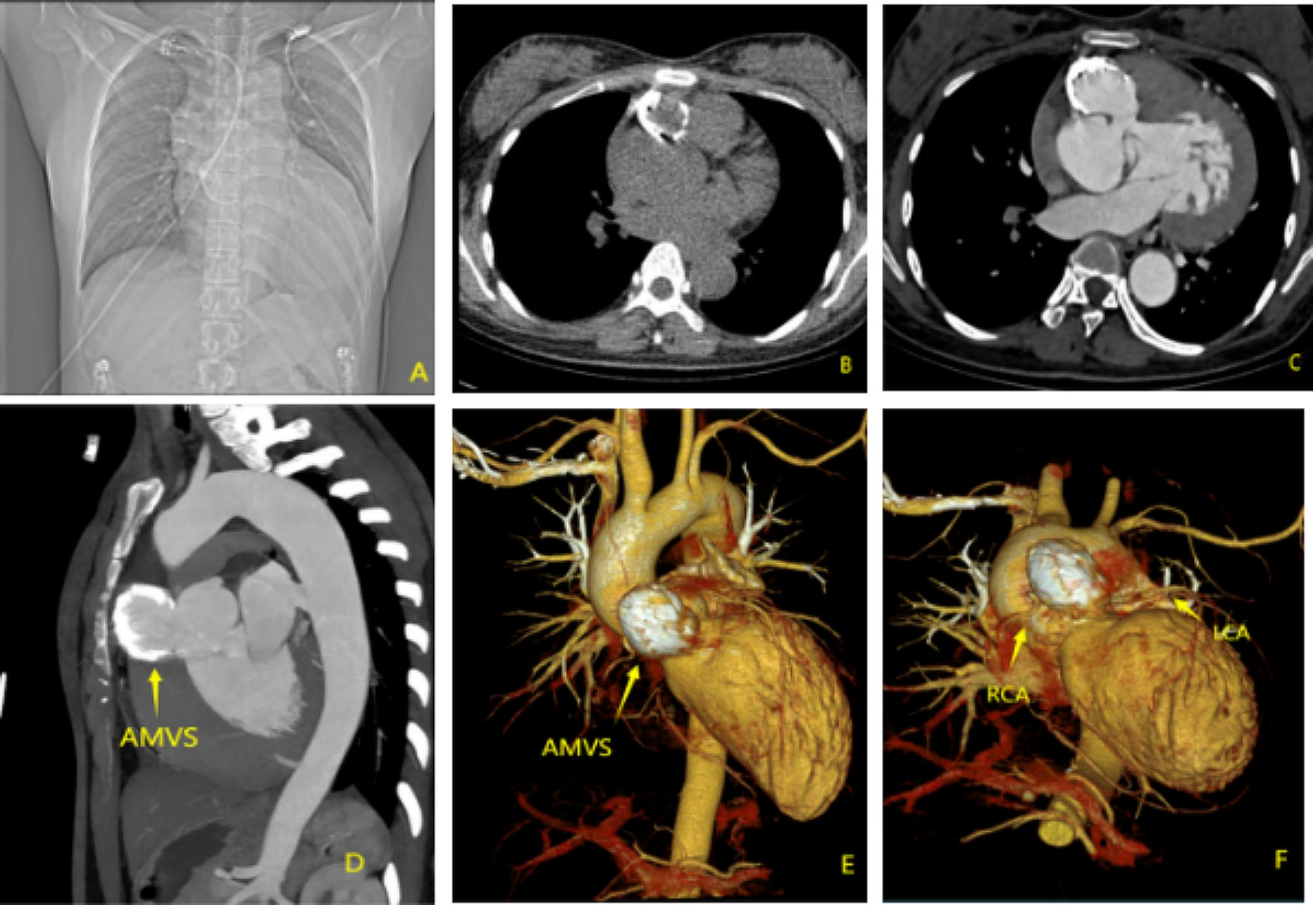
**(A)** Conventional chest x-ray showed significant enlargement of the left heart and widening of the aortic root. **(B)** CT showed widespread calcification of the aneurysm wall. **C, D** Contrast-enhanced CT displayed that the aneurysm was filled with contrast agents and connected to the left ventricular outflow tract. **E.** Three-dimensional CT showed a dilated ascending aorta with a giant aneurysm located below the aortic root. **F.** CTA showed the aneurysm was located close to the right coronary artery

A 22-year-old female was admitted to our hospital for termination of an unwanted pregnancy, she also had become progressively breathless on exertion for six months. At the time of admission, the patient’s general vital signs were good, with a temperature of 36℃, 18 respirations / min, a blood pressure of 131 / 49mmHg, and a heart rate of 91 beats / min. A grade 4/6 systolic murmur and a grade 4/6 diastolic murmur were heard in the aortic valve auscultation area. The patient’s electrocardiogram indicated a complete right bundle branch block with abnormal Q waves in the anterior and inferior walls. TTE indicated a significant dilatation of the aortic annulus and aortic sinus with 46 mm and 58 mm respectively. There was a large membranous ventricular septal defect (MVSD) approximately 14 mm wide in diameter (Fig. 1. A、Video 1). A giant aneurysm, which was about 50 mm×36 mm×26 mm in size, was detected extending from the MVSD to the anterior surface of the aortic root. Color Doppler Flow Imaging (CDFI) showed no significant flow signal entering the aneurysm through the MVSD during systole, whereas bright flow signals generated by eccentric regurgitation of the prolapsed left coronary cusp was observed entering the aneurysm through the MVSD during diastole. The vena contracta width of the eccentric aortic regurgitation was 12 mm, the aortic regurgitation area was 23cm^2^ (Fig. 1. B、Video 2). The peak systolic velocity of the aortic valve flow was 4.56 m/s (peak pressure gradient 83mmHg) (Fig. 1. C). Mild tricuspid regurgitation was observed, and the estimated pulmonary artery systolic pressure was 37mmHg. Contrast-enhanced CT and three-dimensional CT showed a giant aneurysm located below the aortic root and connected to the LVOT. The size of the aneurysm was approximately 30 mm×43 mm×52 mm with widespread mural calcification. (Fig. 2)

The patient underwent aortic valve replacement and aneurysmectomy on 11/22/2023. Intraoperative TEE showed significant dilatation of the aortic annulus and severe regurgitation of the aortic valve. Severe eccentric aortic regurgitation was detected entering the giant aneurysm through the MVSD. (Fig. 1. E、Video 3) The aneurysm was adjacent to the opening of right coronary artery and widespread mural calcification was detected. (Fig. 1. F)

**Fig. 3:**
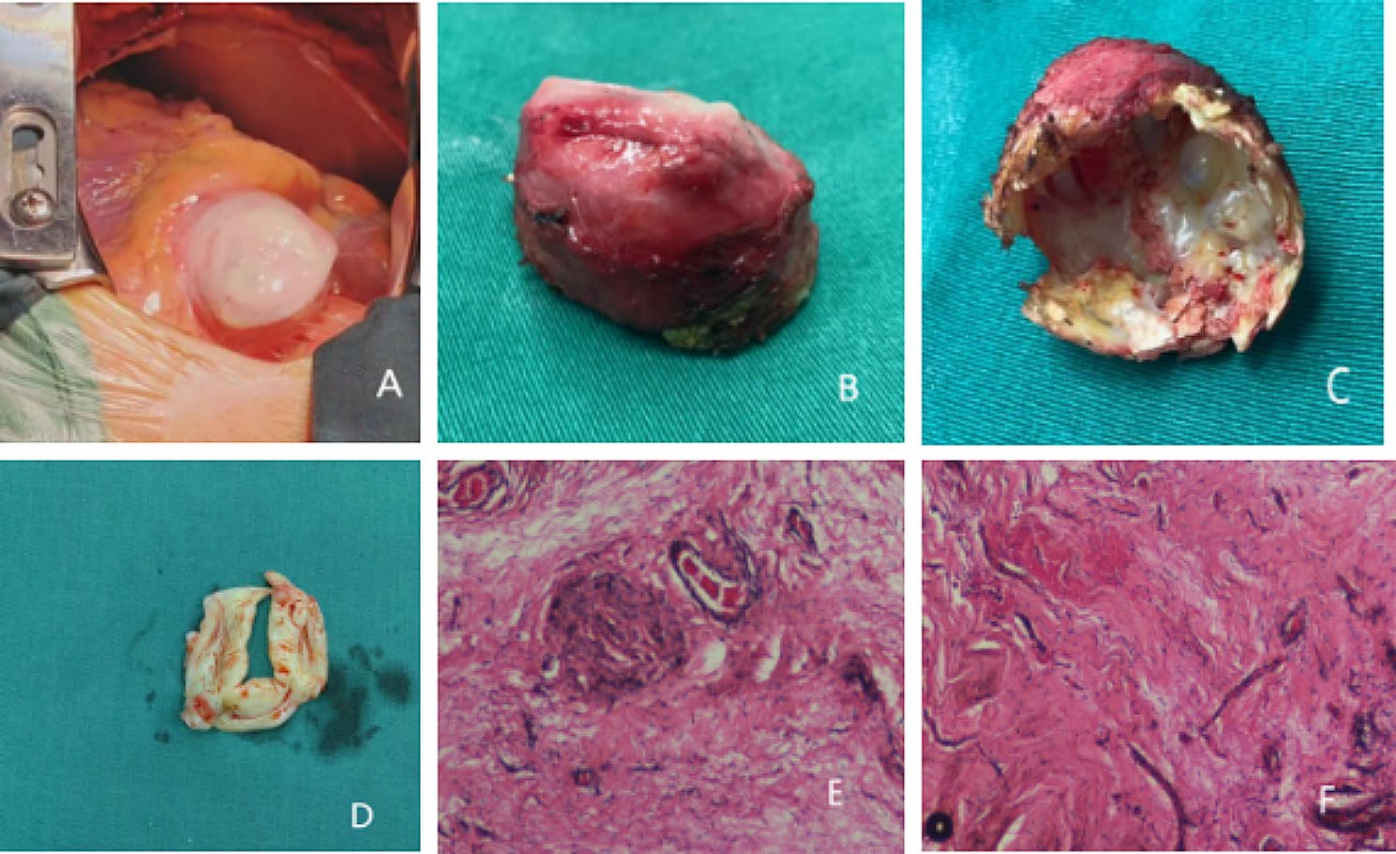
**A.** After opening the chest cavity, a pulsating mass was found protruding from the pericardium. **B, C.** The removed aneurysm had a thick and hard wall with widespread calcification in the inner wall, and the surface was covered with a small amount of pericardium. **D.** Mucous degeneration can be seen in the aortic valve. **E, F.** Microscopic examination showed fibrous connective tissue, adipose tissue, vasodilatation, congestion and lymphocyte infiltration in addition to extensive calcification of the wall. The diagnosis of membranous septal aneurysm was made based on the above pathologic characteristics

Intraoperatively, a giant aneurysm which felt like an iron egg due to its widespread mural calcification was found below the root of the aorta (Fig. 3.A). The aneurysm was removed and sent for pathological examination, which confirmed that in addition to extensive mural calcification, fibrous connective tissue, adipose tissue, vasodilatation, congestion and lymphocytic infiltration were observed, and a diagnosis of a membranous septal aneurysm was made based on the above pathologic features (Fig. 3). The diseased aortic valve was replaced with a bioprosthetic valve since the patient wanted to get pregnant in the future.

The patient recovered well after surgery and was discharged after one week of observation.

## Discussion

Aneurysm of Membranous Ventricular Septum is a less common form of septal disease [1]. According to scholars, AMVS accounts for 0.3% of all congenital heart diseases and 19.1% of VSD [2]. AMVS is formed in different periods from newborns to 6 years, but most often formed in the early stages of infancy [3]. Onat et al. [4] reported that spontaneous closure was strongly associated with AMVS formation, which is seen in 5% of adult patients.

AMVS is always a natural closure of the ventricular septal defect. VSD is subjected to high-pressure blood flow, resulting in endocardial fibroplasia around the VSD with irregular fibroplasia as well as adhesive fusion of the septal valve, the anterior septal valve junction, and some of the tendinous cords. Due to the hemodynamic changes, the morphology of the aneurysm is often altered.

Aneurysms are usually reported as small to medium sized, ranging from 1 to 3 cm in diameter [5]. In this case, the diameter reached an astonishing size of 5 cm. The wall of the aneurysm was thickened irregularly due to the widespread calcification which may be related to prolonged blood flow impingement and an inflammatory response.

The membranous septum originates in part from the endocardial cushion and is close to the heart valves and conduction system. Therefore, the patient may experience ventricular block, which explains the abnormal ECG of the patient.

The main differential diagnosis of MVSD is aortic sinus aneurysm (ASA), also known as coronary sinus aneurysm. On TTE, the ASA is located above the aortic annulus, while the MVSD is located below the aortic annulus. In the present case, the aneurysm started below the aortic annulus, extended across it and ultimately grew along the aortic root. Both TTE and TEE showed no significant blood flow entering the aneurysm during systole, we hypothesized that this might be due to the widespread mural calcification of the wall. Commonly, biphasic flow exists in septal membranous aneurysm. However, in this case, due to the large size and widespread calcification of the aneurysm, the pressure gradient between the aneurysm and the left ventricle was not obvious, so there was no LV-AMVS shunt during systole. During the diastolic period, the pressure in the left ventricle decreased. At this time, there was a large amount of regurgitation of the aortic valve that should have been closed. These regurgitation from the aortic valve entered the aneurysm through the ventricular septal defect, formed eddy currents in the aneurysm, flowed into the left ventricle, and exited with the next cardiac cycle.

It has been shown that the incidence of aortic regurgitation and aortic valve prolapse is significantly higher in patients with AMVS (29% and 47%) than in patients with VSD (4.9% and 20.2%) [5]. Some scholars have reported the incidence of aortic regurgitation in unoperated pediatric or adult patients with VSD to be 5.5 – 18% [6]. Freedom and his colleagues reported aortic valve prolapse in 2 of 32 (6.25%) patients with AMVS [7]. The size of VSD may be an important factor contributing to aortic valve prolapse and regurgitation. A larger VSD can leave the aortic annulus unsupported, leading to secondary aortic annular dilatation.

In a series of studies by Yilmaz [5], AMVS was associated with serious complications. Aneurysm formation functionally reduces the size of the VSD, but has the potential to cause tricuspid valve insufficiency, aortic valve prolapse, right ventricular outflow tract obstruction, aneurysm rupture, and bacterial endocarditis.

In isolated membranous septal aneurysms without residual shunts, the defect diameter and index cross-sectional area of the aneurysm are predictive of prognosis, spontaneous closure and successful surgical closure [8]. Once the diagnosis of AMVS is confirmed, all surgical indications should be treated aggressively to avoid complications such as aneurysm rupture, obstruction, thromboembolism and endocarditis. With the increasing sophistication of surgical techniques such as interventional therapy, the prognosis of the disease is generally favorable.

Coronary CTA is widely used in clinics because of its advantages of being non-invasive and simple [9]. It can clearly show not only the coronary artery but also the location of AMVS, the length and diameter of the aneurysm, the width of the basal portion, whether there is a thrombus in the aneurysm and whether the aneurysm is ruptured. Through multi-temporal reconstruction, the motion of the aneurysm can be observed dynamically, which provides clear and effective image data for clinical intervention in advance.

Echocardiography has a high clinical application value for AMVS. It can dynamically observe the changes of AMVS during each cardiac cycle. CDFI can display the blood flow signals and observe whether there is any blood flow entering AMVS. TTE or TEE should observe the position and size of the AMVS, as well as the relationship between AMVS and its surrounding structures.

### Electronic supplementary material

Below is the link to the electronic supplementary material.

Supplementary Material 1

Supplementary Material 2

Supplementary Material 3

## Data Availability

No datasets were generated or analysed during the current study.
